# Endoscopic Foraminotomy for the Treatment of Lumbar Neuro-Foramen Stenosis: Role of CT in Treatment Planning and Post-Operative Assessment

**DOI:** 10.3390/life15040615

**Published:** 2025-04-07

**Authors:** Giovanni Foti, Gianluca Tripodi, Giuseppe Ocello, Guglielmo Manenti, Giorgio Merci, Thomas Mignolli, Lorenza Sanfilippo, Massimo Guerriero, Gerardo Serra

**Affiliations:** 1 Department of Radiology, IRCCS Sacro Cuore Hospital, 37024 Negrar, Italy; thomas.mignolli@sacrocuore.it; 2 Department of Radiology, Policlinico G. Martino, 98124 Messina, Italy; gianlucatripodi.gt@gmail.com (G.T.); giuseppeocello27@gmail.com (G.O.); 3 Dipartimento di Diagnostica per Immagini e Radiologia Interventistica, Azienda Ospedaliero Universitaria, Policlinico Tor Vergata, 00133 Rome, Italy; gu.manenti@gmail.com; 4 Dipartimento di Biomedicina e Prevenzione, Università di Roma Tor Vergata, 00133 Rome, Italy; 5 Department of Anesthesia and Analgesic Therapy, IRCCS Sacro Cuore Don Calabria Hospital, 37024 Negrar, Italy; giorgio.merci@sacrocuore.it (G.M.); gerardo.serra@sacrocuore.it (G.S.); 6 Clinical Research Unit, IRCCS Sacro Cuore Hospital, 37024 Negrar, Italy; lorenza.sanfilippo@sacrocuore.it (L.S.); mguerriero@univr.it (M.G.)

**Keywords:** CT, foraminotomy, treatment planning, measurement

## Abstract

Purpose: to outline the role of CT in pre- and post-treatment evaluation in the case of lumbar endoscopic foraminotomy. Methods: This prospective study, conducted between September 2020 and January 2024, included consecutive patients with clinical symptoms of lumbar sciatica/lumbalgia/lombo-cruralgia/lower limb peripheral neuropathy. Pre- and post-foraminotomy CT imaging was used to assess the foraminal diameters (cranio-caudal, transverse and free hand ROI area) before and after the treatment. Two independent blinded readers assessed the CT randomly. VAS pain scale and the measurements of each foramen were compared before and after treatment. Interobserver agreement was assessed using the Intraclass Correlation Coefficient (ICC). Results: A total of 47 participants were enrolled, with 53 intervertebral levels analyzed. The mean VAS value decreased from 9.17 in the preoperative period to 0.66 at the one-month postoperative follow-up. The clinical response was associated with statistically significant changes in the cranio-caudal and transverse diameters, as well as the area of the treated neuroforamina (*p*-values < 0.05). Inter-rater reliability between the two operators ranged from 0.75 to 0.90. Conclusions: CT can demonstrate a significant enlargement of the neuroforaminal diameters after the endoscopic foraminotomy, with good correlation with clinical improvement.

## 1. Introduction

Lumbar sciatica is a very common issue in clinical practice [1,2,3]. It is estimated that at least 50% of the population will experience an episode of back pain with or without sciatica at some point in their lives [1,4]. The most common cause of lower back pain with radiation along the course of the sciatic nerve is a herniated disc [4,5,6]. The herniated component, originating from the nucleus pulposus of the disc at the L4–L5 and L5–S1 levels, compresses the nerve roots, causing classic sciatica symptoms [6,7]. However, not all cases of lumbar sciatica are due to disc herniation [8,9]. Indeed, the reduction of the diameters of the bony structures at the vertebral canal and especially the foramina can cause similar and equally debilitating symptoms [10,11,12]. In this case, the pathogenic mechanism is due to the reduction of space available for the nerve roots. Concerning the foramina, inter-apophyseal osteoarthritis—characterized by hypertrophy and thickening of the ligamenta flava—reduces the space available for the sciatic nerve roots [13,14,15,16,17]. When medical therapy and physical rehabilitation fail to resolve this condition, in the case of a herniated disc, the discomfort can be managed by performing a discectomy (open or percutaneous), removing the herniated component causing nerve compression [18,19,20]. Similarly, in the case of foraminal stenosis, the therapeutic rationale is based on decompression of the nerve, achieved by enlarging the foraminal diameters [21,22,23]. Recent percutaneous endoscopic techniques allow targeted enlargements of the foraminal diameters using dedicated instruments (foraminotomy with a drill) [24,25,26]. The location and extent of the enlargement intervention could be appropriately planned using CT or MRI imaging guidance [27,28,29]. MRI has the advantage of greater accuracy in diagnosing herniation and better evaluation of ligament hypertrophy and to rule out the presence of soft tissue lesions that could be missed on CT. In the paper of Heard et al. [15], foraminal stenosis was determined on magnetic resonance imaging and graded using Lee et al.’s validated methodology. The authors demonstrated that regardless of foraminal stenosis severity preoperatively, patients have a similar improvement in PROMs, surgical outcomes, and restoration of motor function after lumbar decompression surgery for radiculopathy [15]. However, MRI may fail to detect subtle bony and soft tissue changes post-operatively, because of the presence of inflammation changes and because of its limited spatial resolution.

On the other hand, computed tomography is undoubtedly the most useful technique for studying the bony structures due to its higher spatial resolution, with additional advantages of short scan times and the possibility of 2D and 3D reconstructions that allow for a more precise evaluation of the foramina [28,29]. Also, recent CT scanners showed an improved contrast resolution, with high accuracy in diagnosing disk herniation and even spondilodyscitis [30,31]. In these settings, preoperative CT could be used to identify the stenotic foramen, confirming the level to be treated; also, CT can be used to demonstrate a significant enlargement of foramen according to the symptomatic outcome, and is important in medico-legal terms to demonstrate the success of the intervention [28,32,33]. Our idea is that, once bone remodeling has been demonstrated, it is evident that there will also be a clear remodeling of the overlying soft tissues. To the best of our knowledge, there are no papers available assessing whether CT can identify, in clinical practice, the presence of symptomatic foraminal stenosis before surgery, or whether it can demonstrate the enlargement of treated foramina in the postoperative assessment. The aim of this study is to outline the role of CT in pre—and post—treatment evaluation in cases of lumbar endoscopic foraminotomy.

## 2. Methods

### 2.1. Study Sample

**Figure 1:**
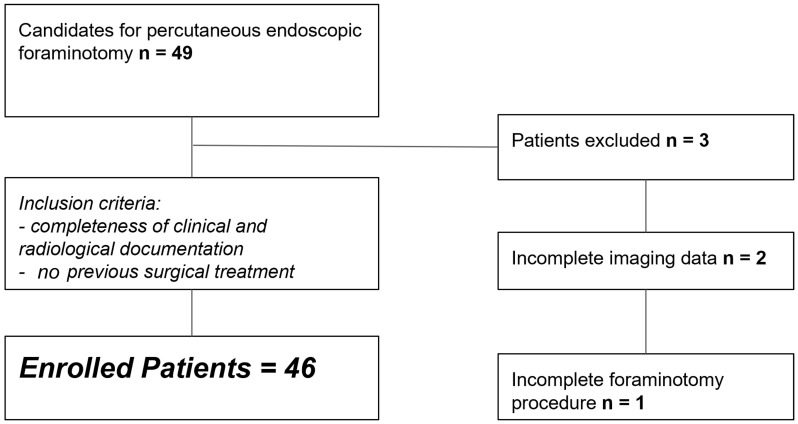
Flowchart of patients enrolled in the study.

This prospective single-center study was approved by the institutional review board (IRB), and informed consent was obtained from all participants (Prot n. 39507; Prog. 2806CESC). Between September 2020 and January 2024, 46 consecutive patients with clinically suspected lumbar foraminal stenosis (during orthopedic or antalgic unit visits) were considered for inclusion. Inclusion criteria were unilateral lumbar pain irradiated to the groin, hip, leg to the foot, and no previous surgery. All patients underwent CT (research examination) within 3 days before and after treatment. All patients were also studied with MRI preoperatively to rule out other possible causes of lumbar or sciatica pain. Exclusion criteria included incomplete imaging data, previous trauma, oncologic disease, and previous vertebral compression fractures [34]. Clinical follow-up was performed for all patients at 1 week and 1 month (Figure 1).

### 2.2. Clinical Evaluation

All patients were clinically assessed before treatment using a dedicated VAS scale ranging from 1 to 10, where 1 represents no pain and 10 represents disabling pain. The levels of pain and sciatica were then recorded [27]. When an overlap between levels did exist, the treatment of multiple levels on the same size was considered. The VAS scale was achieved again at 1 week and 1 month after treatment.

### 2.3. CT Protocol

CT was performed with a third-generation 384-slice dual-source CT scanner (Somatom^®^ Definition Force, Siemens Healthcare, Siemens AG, Erlangen, Germany). The scanning parameters were as follows: Tube A at 80 kV and tube B at 150 kV with a tin filter. The predefined tube current–time product was set at a ratio of 1.6:1 (tube A, 220 quality reference mAs; tube B, 138 quality reference mAs). Automated attenuation-based tube current modulation (CARE dose 4D; Siemens Healthcare, Siemens AG, Erlangen, Germany) was used. The mean effective post-scan volume CT dose index and dose-length product in the leg and in the spine were 9.8 Gy ± 1.9 and 162.5 mGy/cm ± 38 (effective dose 0.03) and 8.2 Gy ± 1.2 and 122.2 mGy/cm (effective dose 0.03) ± 21, respectively.

### 2.4. CT Post-Processing

After each CT examination, the 3D datasets were used for reconstruction (thickness, 0.75 mm; increment, 0.6 mm). The 1 mm axial, sagittal and coronal reconstructed images (Br 64 Kernel—osteo-window filter) were used for clinical reading. The planes were adjusted according to the operator choice to visualize and measure each foramen diameter with a perpendicular approach [24]. 

Soft-tissue kernel (Qr32) images were used for the assessment of disk herniation and ligament hypertrophy.

### 2.5. Image Analysis

Bone windows reconstructed images (1 mm) were employed for the measurement of foramen diameters. The transverse diameter (TD) and Cranio-caudal diameter (CCD) of foramina were measured. Furthermore, the area was measured by using a free hand region of interest (ROI) on the same plane. The measurements were performed before the treatment and after the treatment in each patient at each level for the left and right side. The 2 board certified radiologists were blinded to the clinical scenario (G.F. with 16 years of experience, and EO with 11 years of experience). For each case, each reader chose the preferred slice thickness and windowing settings for the standard and DECT images.

### 2.6. Statistical Analysis

Descriptive statistics, measures of precision and variability, were used to summarize demographic and clinical data.

A Shapiro-Wilk test was performed to assess normality in distribution of continuous variables.

A paired Wilcoxon signed rank test was performed to compare VAS scores at the three time points (pre-treatment vs. 1 week, pre-treatment vs. 1 month, 1 week vs. 1 month).

The Wilcoxon rank sum test was performed to compare measurements of the cranio-caudal and transverse diameters, as well as the area of the neuroforamina at the L3–L4, L4–L5, and L5–S1 levels, both pre- and post-foraminotomy, between the two groups according to symptom status (1 = symptoms present, 0 = symptoms absent). The values were expressed in millimeters (mm) for the cranio-caudal and the transverse diameters, and in square millimeters (mm^2^) for the area.

In addition, for each foramen and for each observer, the delta of the three measurements (computed as the difference between post-treatment and pre-treatment) among the two groups (symptoms present vs. symptoms absent) were compared.

Interrater reliability was assessed using the Intraclass Correlation Coefficient (ICC), with ICC values categorized into four levels of reliability (poor, moderate, good, and excellent) according to the classification framework established.

A *p*-value < 0.05 was considered statistically significant.

Statistical analyses were performed using R Software, version 4.3.0.

### 2.7. Foraminotomy Surgery

**Figure 2:**
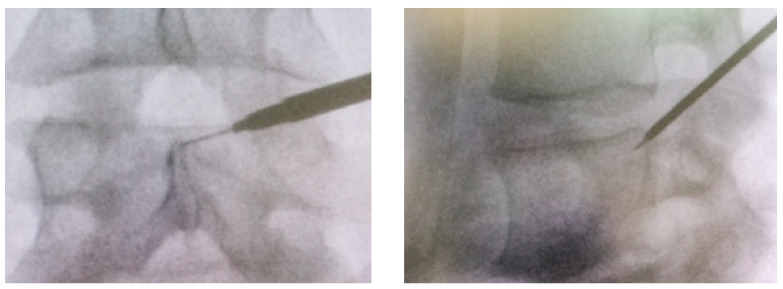
An 18G needle, 150 mm in length, is inserted into the foramen with the target being Kambin’s Triangle, the lower part of the intervertebral foramen. The needle position is confirmed with both anteroposterior (AP; a) and lateral fluoroscopic view.

The patient is positioned prone on the operating table. The foraminotomy itself is divided into two key phases: the first, involving instrument placement under fluoroscopic guidance; and the second, involving the endoscopic procedure. During the first phase, the entry point is determined by marking the spinous process, disc level, and a specific distance from the midline, depending on the spinal level being treated. The determination of the lateral access distance from the spinous process line is influenced by the size of the intervertebral foramen and the patient’s body type. For larger foramina, commonly seen at levels L2/3 and L3/4, an access point about 10 cm from the midline is adequate. At levels L4/5 and L5/S1, a lateral approach positioned 12–14 cm from the midline is typically suitable for a normal-sized foramen. Once defined, an 18G needle, 150 mm in length, is inserted into the foramen with the target being Kambin’s Triangle, the lower part of the intervertebral foramen. The needle position is confirmed with both anteroposterior (AP) and lateral fluoroscopic views (LL) (Figure 2). After the needle is properly positioned, the stylet is removed, and a guide wire is inserted. A scalpel is then used to create a skin incision of approximately 1 cm at the puncture site. The green guiding rod is then placed over the guide wire to the neuroforamen. Three color-coded guiding tubes of increasing diameter are sequentially introduced over the rod to gradually dilate the muscle tissue. If needed, the color-coded reamers are used between the guiding tubes to remove the dorsal-caudal part of the neuroforamen, particularly when the guiding tubes have difficulty reaching the correct position. The crown reamer, designed with specialized teeth to avoid soft tissue entrapment during insertion, is initially rotated counterclockwise until it contacts bone, at which point it is rotated clockwise to begin reaming. Instrument placement and reaming are closely monitored with AP fluoroscopy, ensuring that the reamer does not extend beyond the medial interpedicular line. Following the sequential dilation, the working cannula is introduced.

**Figure 3:**
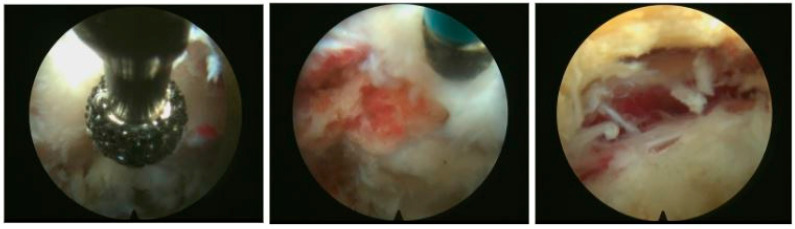
The foraminoscope used for the transforaminal approach has an outer diameter of 6.3 mm, a 30° viewing angle, and a working length of 171 mm. After insertion, soft tissue is cleared to expose the arch of the superior articular process. Once exposed, the shaver system with a 3.5 mm diamond blade is used alternately with the Kerrison rongeur to clear additional bone until the nerve root is visible and decompressed.

In the second phase, the foraminoscope is inserted into the foramen. The foraminoscope used for the transforaminal approach has an outer diameter of 6.3 mm, a 30° viewing angle, and a working length of 171 mm. After insertion, soft tissue is cleared to expose the arch of the superior articular process. Once exposed, the shaver system (operating at 10,000 rpm) with a 3.5 mm diamond blade is used alternately with the Kerrison rongeur to clear additional bone until the nerve root is visible and decompressed. If necessary, ligamentous tissue may also be removed with the Kerrison rongeur to complete the decompression (Figure 3).

## 3. Results

A total of 50 patients were considered for inclusion in our study. Three patients were excluded because of incomplete imaging data (n = 2) and incomplete foraminotomy procedure (n = 1). The study included 47 patients, with a mean age of 71 years, 11 months, and 20 days (age range: 50–91 years), including 27 males and 20 females, all of whom underwent endoscopic foraminotomy. In total, 53 intervertebral levels were treated, as 6 patients received treatment at two distinct levels (Table 1).

All procedures were performed without significant complications, and each intervention was deemed technically successful. The distribution of treated levels was as follows: 11 patients underwent treatment at the right L3–L4 level, 17 at the right L4–L5 level, and 3 at the right L5–S1 level; 1 patient received treatment at the left L3–L4 level, 19 at the left L4–L5 level, and 2 at the left L5–S1 level. Pain intensity was assessed preoperatively, one week postoperatively, and one month postoperatively using the Visual Analog Scale (VAS). Preoperatively, the mean VAS score was 9.17, indicating severe and persistent pain. One week postoperatively, the mean VAS score significantly decreased to 1.96, and at one month, it further decreased to 0.66 (*p* < 0.001). Preoperative CT evaluation revealed that the right L3–L4 level (n = 11) exhibited the most severe stenosis, with the smallest measurements observed (according to reader one’s measurement). This was consistent across both readers. The cranio-caudal diameter (14 mm), transverse diameter (8.73 mm), and foramen area (91.64 mm^2^) were significantly correlated with the clinical symptoms (*p*-value < 0.01).

On the left side, the L4–L5 level (n = 19) was particularly representative, demonstrating similar findings. Here, the cranio-caudal diameter (14.74 mm), transverse diameter (7.63 mm), and area (95.11 mm^2^) also indicated stenosis.

As expected, postoperative CT imaging demonstrated significant increases in foramen dimensions at all treated levels, particularly at the levels previously described. At the right L3–L4 level, post-foraminotomy measurements were 16.26 mm (cranio-caudal), 9.8 mm (transverse), and 115.66 mm^2^ (area). Also, at the left L4–L5 level, a significant widening was achieved, with the cranio-caudal diameter measuring 16.85 mm, transverse diameter 9.34 mm, and area 122.74 mm^2^.

The results before and after the procedure are summarized in Table 2.

**Figure 4:**
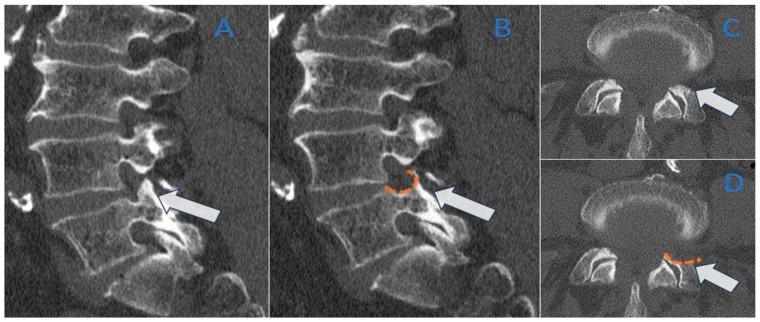
Foraminal stenosis L4–L5; A 57-year-old patient presenting with left lumbar-radicular pain at the L4–L5 level. The preoperative CT images, reconstructed in both sagittal and axial planes with a 1 mm bone window, reveal the presence of a stenotic foramen (arrows), particularly in the postero-inferior aspect, where sclerosis and hypertrophy of the pedicles are evident in images (**A**,**C**). Post-treatment (images (**B**,**D**), dashed orange lines), there is a clear widening of the foramen in the posterior and inferior sectors, with resolution of the hypertrophic and sclerotic components, and a notable recovery of both the foramen area (**B**) and transverse diameter (**D**).

**Figure 5:**
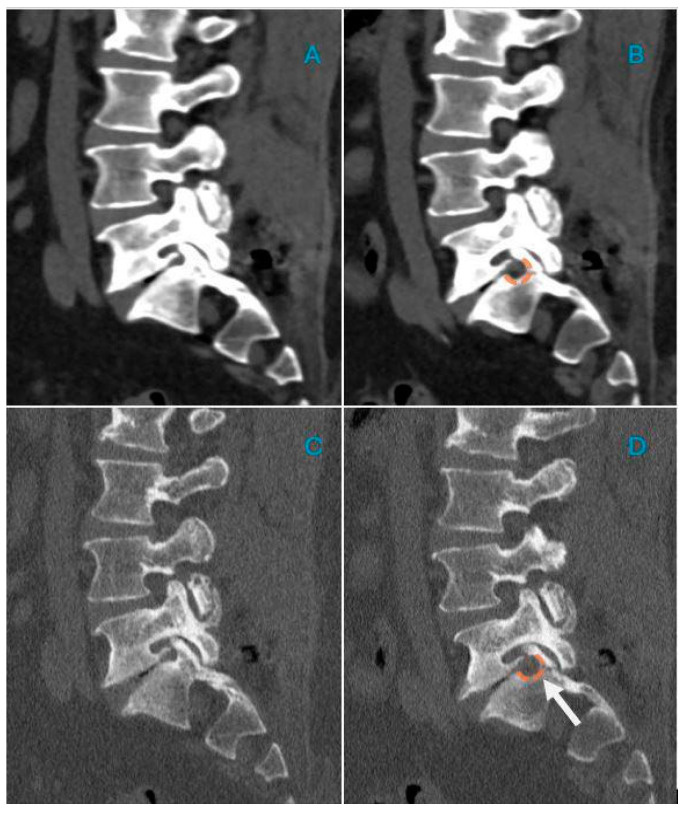
CT sagittal reconstructions for soft tissues (**A**,**B**) and bone (**C**,**D**); a 68-year-old patient presenting with lumbar sciatica. Preoperative images (**A**,**C**) show stenosis of the right L5–S1 foramen, with associated osteophytic formations. Postoperative findings reveal widening of the foramen floor (orange dashed line, images (**B**,**D**)), with persistence of osteophytic spurring, but with an enlargement that mainly involves the inferior and posterior sectors of the foramen (white arrow in (**D**)).

Figure 4 and Figure 5 show two explicative cases before and after treatment.

The ICC values across the various levels of analysis demonstrate an overall good agreement between the two readers in the pre- and post-treatment evaluation of AREA, CC, and TR measurements, with values ranging from 0.094 at L3–L4 on the right side to 0.900 at L3–L4 on the left side. The table provides a detailed presentation of the ICC values along with their confidence intervals between the pre- and post-treatment phases (Table 3).

## 4. Discussion

In this paper, we assessed the role of CT in the evaluation of lumbar neuro-foramens before and after the endoscopic foraminotomy. In particular, the diameters of symptomatic and asymptomatic foramens were compared.

Foraminotomy is a procedure designed to increase the cranio-caudal and transverse dimensions of the neuroforamen, playing a crucial role in the treatment of disc-root stenosis [21,22,23,24,25,26]. This procedure, which can be performed using an open approach, but also through a micro-surgical technique or a fully endoscopic approach, aims to enlarge the foraminal area, helping to alleviate painful symptoms caused by nerve root compression [21,22,23,24,25,26]. The growing application of foraminotomy in clinical settings is driven by its ability to relieve symptoms and improve patients’ quality of life, making it an increasingly sought-after intervention.

As expected, the diameters of treated foramens significantly increased, with good correlation with pain relief. This approach allowed us to establish a correlation between anatomical results and clinical response, creating a basis for evaluating the potential success or failure of the procedure. In our opinion, objective documentation of anatomical changes may represent a tool for quality management.

From a clinical point of view, a significant improvement was achieved in all patients enrolled. VAS scale value decreased from 9.17 at the preoperative period to 0.66 at the postoperative follow-up at one month. Additionally, an improvement was observed as early as one week after the procedure, with a VAS value of 1.96, highlighting the immediacy of the treatment response.

The clinical response was associated with statistically significant changes in cranio-caudal and transverse diameters, as well as the area of the treated neural-foramina (*p*-values < 0.05). For example, at the L3–L4 and L4–L5 levels, cranio-caudal and transverse diameters significantly increased after the procedure (*p*-values were <0.001), confirming the consistency of the data obtained.

The inter-rater reliability between the two operators was good to excellent, allowing reliability and reproducibility for diameters and areas, with ICC values ranging from 0.75 to 0.90. This suggests that the foraminal diameter measurements are consistent and repeatable, allowing for good standardization between different operators.

Additionally, the data suggests a strong concordance between treatment, symptomatology, and CT findings, particularly regarding cranio-caudal diameter and the neuroforaminal area. This indicates not only that the procedure is effective in improving symptoms, but also that there are measurable correlations between anatomical changes and clinical improvement. However, when comparing symptomatic and non-symptomatic foramina in the preoperative phase, a significant difference in foraminal dimensions was only occasionally demonstrated. For instance, a significant difference between the symptomatic and asymptomatic side was achieved only for the right L3–L4 and L4–L5 foramens and for the left L4–L5 one. A possible explanation is the limited number of treated sites at several levels, which prevented reliable calculations. Also, the multifactorial nature of radicular symptoms may play a crucial role. These symptoms, indeed, can depend on herniated discs and other factors related to vertebral instability, both of which were not evaluated in this study. For this reason, we did not attempt to establish specific cutoffs to distinguish symptomatic from asymptomatic foramina, as the decision to proceed with this therapy largely depends on the clinical context. However, the evaluation of foraminal diameters using CT—performed, for example, during pre-procedural planning or as part of imaging workup—can be essential for obtaining an accurate assessment of the actual diameters of each foramen. This can lay the groundwork for a useful follow-up, demonstrating any further reduction in the bony foraminal diameters over time.

The study by Evins AI et al. [28] has already demonstrated the potential for significantly expanding foraminal dimensions. In their cadaveric research, 48 transforaminal endoscopic foraminotomies were performed at the L3–L4, L4–L5, and L5–S1 levels on eight cadavers. CT scans were acquired using a mobile fluoroscopic system, with sagittal images centered on adjacent pedicles to measure foraminal height and area. The results showed that endoscopic foraminotomy led to substantial improvements in foraminal dimensions, with height increasing by approximately 14 mm and foraminal area enlarging by around 19 mm^2^.

Our findings are consistent with these results. Specifically, our measurements revealed increases in cranio-caudal (CC) and transverse (TR) diameters of about 2 mm, along with a foraminal area expansion of approximately 10 mm^2^. However, a direct comparison with Evins et al.’s study is not entirely feasible due to differences in imaging equipment and, more importantly, because our series focused on symptomatic patients. In our cases, the procedure was tailored to achieve clinical improvements while minimizing the risk of complications.

Despite these encouraging outcomes, certain limitations must be acknowledged. First, the absence of standardized measurement criteria poses a challenge, as variations in methodology can affect results. While some grading systems for lumbar stenosis have been proposed [29], our study did not aim to classify stenosis severity, but rather to conduct precise evaluations of CC and TR diameters and foraminal area, consistent with previous research [28].

Additionally, the patient sample in our study was relatively limited and may not fully represent the broader population, potentially limiting the generalizability of the findings. Specifically, we found a high mean VAS of 9.17 before surgery; these procedures are typically reserved at our institution for patients with severe, chronic pain, who have already failed multiple treatments, often including therapies from other centers, and who have not achieved adequate pain relief through medical management. Also, we missed post-procedural data regarding the patients’ medical therapy after the intervention.

Furthermore, overlapping conditions, such as herniated discs and the presence of neurological symptoms caused by multi-level nerve root involvement, complicate pinpointing the specific level responsible for the symptoms, and for this reason, a binary approach was used in the assessment of post-treatment symptoms. The lack of treatment proposals represents another limitation. Finally, only a short follow-up is available, which could limit the clinical impact of our results. Nonetheless, given the excellent initial outcomes and the primary aim of the study being to demonstrate bone enlargement, we consider a short-term follow-up to be adequate.

## 5. Conclusions

In conclusion, endoscopic foraminotomy has proven to be an effective intervention for disc-radicular stenosis, achieving significant improvements in foraminal dimensions and symptom relief. CT has proven to be a reliable method for preoperative planning and for demonstrating foraminal widening after treatment, underscoring a potential relationship between anatomical changes and pain reduction.

## Figures and Tables

**Table 1:** Patients included and intervertebral levels treated.

Characteristic	Total
Sex	
Male	27
Female	20
Mean Age (Range) years	71 (50–91)
Operative Level Left	
L3–L4	1
L4–L5	19
L5–S1	2
Operative Level Right	
L3–L4	11
L4–L5	17
L5–S1	3
Evaluation time	Vas value, mean (SD)
Presurgica	9.17 (0.58)
1 Week	1.96 (1.09)
1 Month	0.66 (0.71)

**Table 2:** Mean and SD value (in parenthesis) of the DELTA between post- and pre-foraminotomy diameters and area at each level, as measured by the two readers; the relative *p*-values from the comparison between symptomatic and asymptomatic side are shown on each level. * = in mm; ** = in squared mm.

Levels	R1 Overall	R1 Symptoms	R1 Not-Symptoms	R1 *p*-Value	R2 Overall	R2 Symptoms	R2 Not-Symptoms	R2 *p*-Value
Left								
L3–L4 (n = 1)								
* cc	0.55 (2.15)	4.00 (0.0)	0.48 (2.12)	0.069	0.02 (1.13)	4.00 (0.0)	0.10 (1.00)	0.001
* tr	0.08 (1.09)	0.00 (0.0)	0.08 (1.10)	>0.9	0.15 (1.76)	0.00 (0.0)	0.15 (1.78)	>0.9
** area	3.96 (14.2)	30.00 (0.0)	3.46 (13.9)	0.064	2.38 (10.7)	40.00 (0.0)	1.65 (9.43)	0.043
L4–L5 (n = 19)								
cc	1.30 (2.36)	3.11 (1.49)	0.29 (2.15)	<0.001	1.21 (2.09)	2.89 (2.21)	0.26 (1.29)	<0.001
tr	1.25 (1.69)	2.26 (1.48)	0.69 (1.55)	<0.001	1.28 (1.82)	2.26 (2.08)	0.74 (1.42)	0.006
area	22.04 (31.4)	48.47 (24.6)	7.26 (24.5)	<0.001	15.81 (18.6)	29.9 (16.4)	7.94 (14.8)	<0.001
L5–S1(n = 2)								
cc	0.30 (0.87)	2.00 (0.00)	0.24 (0.81)	<0.001	0.21 (0.74)	1.50 (0.71)	0.16 (0.70)	0.002
tr	0.68 (2.35)	5.00 (0.00)	0.51 (2.23)	<0.001	0.62 (1.97)	1.50 (4.95)	0.59 (1.88)	>0.9
area	8.23 (16.9)	56.00 (24.0)	6.35 (13.80)	<0.001	5.58 (16.4)	40.0 (46.6)	4.24 (13.6)	0.006
Right								
L3–L4 (n = 11)								
cc	0.66 (1.3)	2.09 (1.58)	0.29 (1.02)	<0.001	0.66 (2.13)	2.91 (4.01)	0.07 (0.34)	<0.001
tr	0.51 (1.9)	2.64 (1.43)	0.05 (1.68)	<0.001	0.75 (1.30)	2.73 (1.42)	0.24 (0.58)	<0.001
area	10.96 (21.3)	35.73 (19.8)	4.48 (16.6)	<0.001	10.6 (21.6)	34.8 (23.1)	3.57 (15.9)	<0.001
L4–L5 (n = 17)								
cc	1.72 (2.03)	3.29 (1.99)	0.97 (1.59)	<0.001	2.40 (2.96)	4.24 (2.68)	1.53 (2.71)	0.001
tr	1.57 (2.05)	3.12 (1.54)	0.83 (1.86)	<0.001	1.17 (1.92)	2.35 (2.37)	0.61 (1.38)	0.001
area	25.06 (24.2)	43.24 (11.4)	16.47 (24.0)	0.001	22.1 (24.80)	39.3 (20.6)	14.06 (22.5)	<0.001
L5–S1 (n = 3)								
cc	0.09 (0.97)	1.33 (3.51)	0.02 (0.62)	0.15	0.04 (1.45)	2.00 (5.29)	0.16 (0.91)	0.2
tr	0.06 (0.60)	0.67 (1.15)	0.02 (0.55)	0.2	0.09 (0.65)	1.33 (1.53)	0.02 (0.50)	0.019
area	2.26 (11.50)	24.33 (18.0)	0.94 (9.77)	0.003	0.96 (11.9)	29.3 (31.3)	0.74 (7.71)	0.001

**Table 3:** Table shows the ICC values for the L3–L4, L4–L5, and L5–S1 foramina on both the right (DX) and left (SX) sides for the three measurements (CC, TR, and AREA) in both the pre- and post-treatment phases, with the confidence interval values reported in parentheses.

		L3 L4 DX	L4 L5 DX	L5 S1 DX	L3 L4 SX	L4 L5 SX	L5 S1 SX
Pre-treatment	CC	0.749 (0.586–0.851)	0.633 (0.419–0.776)	0.769 (0.631–0.859)	0.900 (0.834–0.941)	0.471 (0.237–0.655)	0.851 (0.754–0.912)
	TR	0.300 (0.032–0.527)	0.630 (0.434–0.769)	0.332 (0.065–0.554)	0.730 (0.528–0.845)	0.503 (0.273–0.679)	0.460 (0.179–0.662)
	AREA	0.905 (0.841–0.944)	0.595 (0.387–0.745)	0.889 (0.792–0.939)	0.509 (0.281–0.683)	0.667 (0.488–0.793)	0.807 (0.554–0.906)
Post-treatment	CC	0.733 (0.551–0.844)	0.890 (0.817–0.935)	0.772 (0.636–0.861)	0.631 (0.439–0.768)	0.859 (0.745–0.920)	0.848 (0.751–0.909)
	TR	0.094 (-0.183–0.357)	0.550 (0.332–0.712)	0.401 (0.121–0.615)	0.427 (0.180–0.623)	0.490 (0.257–0.670)	0.678 (0.408–0.823)
	AREA	0.849 (0.752–0.910)	0.680 (0.504–0.802)	0.817 (0.700–0.890)	0.402 (0.154–0.603)	0.847 (0.750–0.909)	0.847 (0.700–0.918)

## Data Availability

The data presented in this study are available on request from the corresponding author. The data are not publicly available due to Hospital policy.
